# Analysis of the diagnostic role of fibrin-related markers in perioperative venous thromboembolism

**DOI:** 10.3389/fsurg.2025.1530576

**Published:** 2025-05-20

**Authors:** Min Wei, Yongjiang Pan, Zheng Qin, Lihua Chen, Liping Zhao, Debin Zhong

**Affiliations:** Medical Laboratory Department, The First People's Hospital of Nanning, Nanning, China

**Keywords:** venous thromboembolism, fibrin-related markers, perioperative period, diagnosis, surgery

## Abstract

**Objectives:**

To investigate the diagnostic role of fibrin-related markers in the perioperative venous thromboembolism (VTE).

**Methods:**

A total of 100 high-risk perioperative patients, identified using the Caprini thrombosis risk assessment model [46 with deep vein thrombosis (DVT) and 54 with pulmonary embolism (PE)], were included as study subjects. Additionally, 50 healthy volunteers undergoing medical checkups during the same period served as controls. The levels of D-dimer, fibrin monomer (FM) and fibrin degradation products (FDP) were compared between patients and the control group across different time points. Additionally, the influence of various factors on these biomarkers was assessed. Furthermore, the diagnostic value of fibrin-related markers in perioperative VTE among surgical patients was analyzed.

**Results:**

The levels of D-dimer, FM and FDP on postoperative days 1, 3 and 7 were significantly lower than those on the day before surgery, showing a sequential decline over the postoperative period (*p* < 0.05). The levels of these three markers were significantly higher in the PE group than in the DVT group, and higher in the DVT group than in the control group (*p* < 0.05). No significant differences were observed in these markers based on gender, age or disease conditions (*p* > 0.05). Additionally, ROC curve analysis indicated that FM detection alone had good diagnostic value, with an AUC of 0.835 and a sensitivity of 88.34%. And joint detection (D-dimer, FM, FDP) provided superior diagnostic performance.

**Conclusion:**

The combination of D-dimer, FM, FDP and Caprini scores may enhance the diagnostic accuracy of VTE in the perioperative period. If all four indicators are not readily available, monitoring FM levels alone can provide valuable diagnostic insight.

## 1 Introduction

Venous thromboembolism (VTE) is a prevalent clinical condition associated with significant morbidity and has become an increasingly common peripheral vascular disease. Epidemiological data indicate that approximately 2.5 million cases of VTE are diagnosed worldwide each year, with at least 0.1% of affected patients succumbing to PE (1, 2). Only 20% of patients with suspected VTE receive a confirmed diagnosis (3). VTE is primarily caused by cancer, surgery and other conditions associated with Virchow's triad: endothelial injury, venous stasis, and hypercoagulability (4). Surgery can act as a VTE risk factor, depending on the type of surgery and patient factors (5, 6). Although advancements in surgical techniques, early ambulation and pharmacologic prophylaxis have reduced postoperative VTE rates (7), VTE remains a leading cause of preventable death in perioperative patients. Therefore, early detection and diagnosis of VTE are critical for patient's prognosis and treatment. VTE diagnosis in surgical patients generally follows a stepwise process including clinical pretest probability, D-dimer testing and imaging confirmation (3, 8). However, imaging is not always necessary, as it is time-consuming, costly, and carries risks such as radiation exposure (9). While D-dimer is a sensitive marker which excluds VTE when negative (10, 11), it lacks specificity, as various conditions (such as surgery, cancer and cardiovascular diseases) can elevate its levels. Both FDP and D-dimer levels rise due to secondary fibrinolysis in VTE, making the combined detection of both biomarkers useful for diagnosis (12). The diagnosis of DVT appears to be more accurate when both FM and D-dimer concentrations are assessed in combination, rather than individually (13–15). And the Caprini score remains one of the most widely used VTE risk assessment tools for surgical patients (16). This study expanded on this diagnostic framework by evaluating the role of fibrin-related markers (D-dimer, FM, FDP) with Caprini score in perioperative VTE. We hypothesized that combining fibrin-related markers (D-dimer, FM and FDP) with Caprini score can improve the diagnostic accuracy of VTE in the perioperative period.

## 2 Data and methods

### 2.1 Clinical data

From January 2020 to December 2021, a total of 100 perioperative patients at high risk for VTE (Caprini score 3–4) were enrolled from the First People's Hospital of Nanning City. All VTE patients met the clinical diagnostic criteria, with DVT confirmed by venography and ultrasound, and PE confirmed by CT venography. Patients were required to be aged 20 years or older, and informed consent was obtained from their families. Patients with severe organ dysfunction, those who were pregnant or lactating, and those with incomplete data were excluded. Among the 100 patients, 54 were male and 46 were female, with 51 patients aged ≥60 years and 49 aged <60 years. The cohort included 46 cases of DVT, designated as the DVT group, and 54 cases of PE, designated as the PE group. Additionally, 50 healthy volunteers undergoing routine physical examinations during the same period were included as the control group, comprising 27 males and 23 females, with 22 aged ≥60 years and 28 aged <60 years. No significant differences were observed between the patient and control groups (*p* > 0.05). This study was approved by the hospital's ethics committee.

### 2.2 Research method

For all subjects, 5 ml of fasting venous blood was collected and then centrifuged at 3,000 rpm/min for 15 min using a centrifuge with a radius of 10 cm to separate the plasma. The levels of D-dimer, FM and FDP in the plasma were subsequently measured using the French Stago fully automated coagulation analyzer and its associated reagent kit. Testing procedures strictly adhered to the step-by-step instructions provided in the reagent kit manual. All the samples were analyzed by STA-Evolution (Stago, France) using STA-R series reagents according to the manufacturer protocol.

### 2.3 Observation indicators

The levels of D-dimer, FM and FDP were compared across different perioperative periods and among different groups. Additionally, the impact of various factors on these biomarkers was analyzed to assess their diagnostic value for venous thromboembolism in perioperative period.

### 2.4 Statistical methods

The data were analyzed using SPSS 23.0 software. Quantitative data are presented as mean ± standard deviation $\left(\bar{x}\pm s\right)$, and comparisons were performed using the *t*-test. Comparison among multiple groups was conducted using analysis of variance (ANOVA), with the F value calculated. The diagnostic role of fibrin-related biomarkers in the perioperative period of VTE was assessed using ROC curve analysis. A *p* value of less than 0.05 was considered statistically significant.

## 3 Results

### 3.1 Comparison of levels of D-dimer, FM and FDP at different periods in patients

The levels of D-dimer, FM and FDP in patients at different time points showed statistically significant differences (*p* < 0.05). The levels of these markers on postoperative days 1, 3 and 7 were lower than those on preoperative day 1, with levels on postoperative days 3 and 7 being lower than those on postoperative day 1. Furthermore, the levels on postoperative day 7 were lower than those on postoperative day 3 (*p* < 0.05), as shown in Table 1.

**Table 1:** Comparison of D-dimer, FM and FDP levels in patients at different time periods (mean±s).

Time	Number of cases	D-dimer (mg/L)	FM (mg/L)	FDP (mg/L)
Pre-op day 1	100	1.91 ± 0.35	11.58 ± 0.61	23.52 ± 4.60
Post-op day 1	100	1.70 ± 0.33*	9.21 ± 0.45*	20.08 ± 3.57*
Post-op day 3	100	1.03 ± 0.24*^,^ **	7.94 ± 1.33*^,^ **	14.39 ± 4.12*^,^ **
Post-op day 7	100	0.68 ± 0.19*^,^ **^,^ ***	4.20 ± 1.08*^,^ **^,^ ***	6.79 ± 0.83*^,^ **^,^ ***
*F* value	–	24.314	59.827	114.261
*P* value	–	0	0	0

Compared with preoperative 1 day, *P < 0.05; compared with postoperative 1 day, **P < 0.05; compared with postoperative 3 days, ***P < 0.05.

### 3.2 Comparison of the levels of D-dimer, FM and FDP at different time periods in each group

The levels of D-dimer, FM and FDP in the PE group at different time periods are higher than those in the DVT group and the control group, with the DVT group also showing higher levels than the control group (*p* < 0.05), as shown in supplemental tables (Supplementary Table S1).

### 3.3 Comparison of the impact of different factors on the levels of D-dimer, FM and FDP in patients

No significant differences were observed in the influence of gender, age, and disease conditions on the levels of D-dimer, FM and FDP in patients (*p* > 0.05), as shown in Table 2.

**Table 2:** Comparison of the impact of different factors on the levels of D-dimer, FM and FDP in patients.

Factor	Number of cases	D-dimer (mg/L)	FM (mg/L)	FDP (mg/L)
Gender				
Male	54	2.03 ± 0.51	11.39 ± 0.35	25.36 ± 3.19
Female	46	2.06 ± 0.47	11.32 ± 0.40	25.40 ± 3.22
Age				
≥60	51	2.12 ± 0.38	11.28 ± 0.51	25.32 ± 4.08
<60	49	2.09 ± 0.50	11.31 ± 0.47	25.30 ± 3.53
Body mass index				
≥20	43	2.19 ± 0.36	10.97 ± 0.62	25.41 ± 4.11
<20	57	2.20 ± 0.26	11.23 ± 0.58	25.39 ± 3.76

### 3.4 Analysis of the diagnostic role of fibrinrelated markers in the perioperative venous thromboembolism

According to the ROC curve analysis, in addition to the joint detection (the level of D-dimer, FM and FDP) and FM detection alone also demonstrated good diagnostic value, with an AUC of 0.835 and a sensitivity of 88.34%, as shown in Table 3.

**Table 3:** Analysis of the diagnostic role of fibrin-related markers in perioperative venous thromboembolism.

Item	AUC	Sensitivity	Youden	Index	Cutoff value	95%CI
D-dimer	0.632	82.49	79.76	0.623	2.03 mg/L	0.418–0.827
FM	0.829	88.27	85.44	0.737	11.94 g/L	0.705–0.874
FDP	0.704	79.56	81.29	0.609	25.38 mg/L	0.627–0.913
Caprini score	0.829	88.27	85.44	0.737	5 points	0.705–0.874
Combined four items	0.917	95.29	83.16	0.785	–	0.790–0.998

## 4 Discussion

In clinical practice, VTE is a common cardiovascular complication and its clinical manifestations are usually lacking in specificity. Symptoms such as fever, difficulty breathing, lower limb swelling or fainting are frequently observed, but the condition is typically insidious and complex. Relying solely on symptoms makes it difficult to establish a clear diagnosis, increasing the likelihood of missed or incorrect diagnoses (17–19). Therefore, early diagnosis and differentiation of VTE clinical symptoms are crucial for improving patient prognosis.

The results of this study show that the levels of D-dimer, FM and FDP in patients at different time points were statistically significant (*p* < 0.05). The levels of these markers on postoperative days 1, 3, and 7 were lower than those on preoperative day 1, and the levels on postoperative days 3 and 7 were lower than those on postoperative day 1. Additionally, the levels on postoperative day 7 were lower than those on postoperative day 3 (*p* < 0.05). These findings indicate that the levels of D-dimer, FM, and FDP in VTE patients peaked on preoperative day 1 and then progressively decreased, reaching their lowest levels on postoperative day 7. Furthermore, the study found that the levels of D-dimer, FM, and FDP in the PE group were higher than those in the DVT group and the control group, with the DVT group showing higher levels than the control group (*p* < 0.05). This suggested that the expression of D-dimer, FM and FDP was significantly elevated in the thrombosis patient population, likely due to the underlying physiological mechanisms associated with these markers. D-dimer is an indirect marker of fibrinolysis and fibrin turnover, reflecting both hemostatic abnormalities and intravascular thrombosis. As a soluble fibrin degradation product, it results from the breakdown of thrombi via fibrinolysis. However, D-dimer lacks specificity, as its levels can be elevated in conditions such as inflammation and infection. Despite this, it remains a valuable marker for coagulation and fibrinolysis activation particularly in ruling out VTE (8, 20). In our study, elevated preoperative D-dimer levels were observed likely due to the high VTE risk as indicated by the Caprini score. Factors such as advanced age, obesity, and comorbidities (like cancer, COPD, infection) may induce systemic inflammation and increased fibrin activity, leading to elevated D-dimer levels. These factors should be considered when interpreting preoperative D-dimer results, as they can raise levels independently of thrombus formation. The diagnostic process for suspected venous thromboembolism involves a stepwise approach that includes the evaluation of clinical pretest probability, D-dimer testing and imaging. Ideally, this work-up should be completed within 24 h of presentation. The goal is to identify patients who require anticoagulation therapy (to confirm VTE) and those in whom imaging and anticoagulation can be safely omitted (to exclude VTE). The combination of clinical pretest probability and D-dimer testing is useful for excluding VTE; however, imaging is necessary to definitively confirm the diagnosis (9). Our study highlighted the potential of combining fibrin-related biomarkers (D-dimer, FM and FDP) with the Caprini score in diagnosing perioperative VTE. While D-dimer has long been recognized as a sensitive biomarker for VTE, its lack of specificity remains a limitation, as elevated levels can be observed in various clinical conditions including surgery, cancer, and cardiovascular diseases (10). Hasegawa et al. reported that FM as a fibrin monomer differs from D-dimer, which is better at reflecting the early stage of thrombus formation. As a precursor in thrombus production, FM is upstream of fibrin degradation products and represents the initial stage of fibrin clot formation during hemostasis. It is formed when thrombin acts on fibrinogen, cleaving it into two peptides and providing valuable feedback on the condition of VTE patient (21–24). Based on the literature reviewed, the diagnosis of DVT seems to be more accurate when both FM and D-dimer concentrations are used in combination rather than when assessed individually (13–15). FDP is an important indicator of hyperfibrinolysis in the body. Their levels increase during primary or secondary fibrinolysis, and in conditions of high blood coagulation or the occurrence of VTE, the levels further rise, which can serve as a prognostic marker (25). FDP level has been shown to correlate with a higher incidence of VTE in patients with gastric and cervical cancers (26, 27). Jiao et al. conducted an analysis of 488 patients with spinal fractures and found that plasma FDP levels ≥5.19 mg/L are a significant risk factor for VTE (28). Furthermore, a study of 569 patients with femoral and pelvic fractures confirmed that elevated FDP levels are associated with a higher incidence of VTE in the perioperative period (29). Therefore, it can be concluded that FDP is a key factor in the development of VTE. Patients with sepsis had significantly higher levels of d-dimer and FPD activity compared with healthy controls, and FM had less studied in sepsis (30). Plasma levels of FDP, D-dimer, and FM are elevated in patients with disseminated intravascular coagulation (DIC). The combination of FM, D-dimer, and FDP has become the gold standard for DIC diagnosis, as recognized by the International Society on Thrombosis and Hemostasis (ISTH). In patients with malignant tumors, a hypercoagulable state is often present, typically accompanied by coagulation and fibrinolysis abnormalities. These patients frequently exhibit elevated plasma D-dimer and FDP levels, which may be closely associated with tumor infiltration. Higher levels of D-dimer and FDP are generally correlated with poorer prognosis in cancer patients (10, 15, 20, 31, 32). A study examining normal pregnancy and early DIC stages found that D-dimer levels increase progressively with gestational age, while FM remains relatively stable during the second and third trimesters. In the early stages of DIC, FM levels significantly rise, and dynamic monitoring of FM may aid in the early detection of DIC (15, 33). Additionally, a study including 120 chronic kidney disease (CKD) patients (stages 2–5), first-time hemodialysis (HD) patients, and maintenance HD patients revealed significantly higher levels of prothrombin complex and D-dimer in CKD patients compared to the healthy population, with the elevated D-dimer levels being particularly pronounced in HD patients (34). Anticoagulant drugs are the most commonly used oral anticoagulant drugs in clinical practice, and both of them can reduce D-dimer levels. It is the anticoagulant effect of the drugs that reduces the activation of the coagulation system and the fibrinolytic system, which indirectly leads to a decrease in the D-dimer level (35). Our findings are consistent with previous studies, which also highlighted the role of fibrin-related biomarkers in VTE diagnosis and extended these findings by incorporating FM and FDP into the diagnostic approach. After surgery, as the patient's clinical symptoms improved, the levels of D-dimer, FM and FDP gradually decreased. The levels in the PE group were significantly higher compared to those in the DVT group. Furthermore, this study found no significant influence of gender, age, or disease conditions on the levels of D-dimer, FM and FDP in patients (*p* > 0.05), suggesting that the changes in these markers were not correlated with the patients' gender, age, or disease conditions. This lack of correlation could be attributed to the fact that thrombosis status is not closely related to these characteristics, and may instead reflect differences in disease characteristics and other factors. Finally, according to the ROC curve analysis, in addition to joint detection, FM detection alone demonstrated significant diagnostic value, with an AUC of 0.835 and 88.34% sensitivity. This indicates that while the combination of D-dimer, FM, FDP and Caprini score may provide better diagnostic accuracy for surgical perioperative VTE, FM detection alone also may offer valuable diagnostic insights. In practice, the comprehensive application of these four indicators can effectively reduce the risk of missed or insufficient diagnoses, providing a more accurate reflection of the patient's condition. If clinical results for all four indicators are not available in a timely manner, monitoring FM levels in advance can still yield significant diagnostic value, thereby serving as a useful adjunct in clinical diagnosis and treatment (36, 37). The combination of D-dimer with FM and FDP could enhance diagnostic accuracy, particularly when clinical symptoms are ambiguous or when other conditions may interfere with interpretation. By integrating biomarkers with the Caprini score, we offered a more objective and quantifiable diagnostic approach compared to solely relying on clinical evaluation or individual biomarker testing. In addition, the use of clinical pre-test probability assessments alongside D-dimer testing has proven effective in ruling out VTE, the definitive diagnosis still requires imaging confirmation (3, 8). The integration of fibrin-related biomarkers with clinical evaluation can further reduce unnecessary imaging, which is costly, time-consuming and associated with risks such as radiation exposure and contrast-induced nephropathy (9). This is particularly critical in the perioperative setting where timely and safe diagnosis is crucial for perioperative patient management. Although the traditional diagnostic imaging remains the gold standard for VTE diagnosis, minimizing unnecessary imaging by incorporating clinical assessments with biomarkers like FM and D-dimer offers significant potential for enhancing patient care. Our findings may highlight specific biomarkers (D-dimer, FM, and FDP) that could enhance early diagnosis and help clinical decision-making. By incorporating these markers, we hope to reduce unnecessary imaging and ultimately improve patient outcomes. This approach could also help streamline the diagnostic workflows, which ultimately can reduce healthcare costs and improve patient outcomes.

However, there are several limitations in our study. First, as a single-center study with a small sample size, the generalizability of our findings may be limited. While the results are promising, larger and multi-center studies are needed to validate and confirm these findings in diverse populations and various healthcare settings. Moreover, although we controlled for key variables, other factors such as the type of surgery, medications and underlying health conditions could influence biomarker levels. Future research should investigate how these variables might affect the utility of biomarkers in diagnosing VTE. Another limitation is the lack of stratification by surgery type and VTE severity. Stratifying patients based on these factors could provide valuable insights into how the performance of biomarkers varies in different surgical contexts and in relation to the severity of VTE.

In conclusion, the combination of D-dimer, FM, FDP and Caprini score can enhance the diagnostic accuracy of VTE in the perioperative period. If the results for all four indicators are not readily available, monitoring FM levels in advance can still provide valuable diagnostic information and improve diagnostic outcomes.

## Data Availability

The original contributions presented in the study are included in the article/Supplementary Material, further inquiries can be directed to the corresponding authors.
